# Nicotine Promotes Acquisition of Stem Cell and Epithelial-to-Mesenchymal Properties in Head and Neck Squamous Cell Carcinoma

**DOI:** 10.1371/journal.pone.0051967

**Published:** 2012-12-26

**Authors:** Michael Andrew Yu, Alan Kiang, Jessica Wang-Rodriguez, Elham Rahimy, Martin Haas, Vicky Yu, Lesley G. Ellies, Jing Chen, Jian-Bing Fan, Kevin T. Brumund, Robert A. Weisman, Weg M. Ongkeko

**Affiliations:** 1 Division of Otolaryngology-Head and Neck Surgery, Department of Surgery, University of California San Diego, La Jolla, California, United States of America; 2 Veterans Administration Medical Center and Department of Pathology, University of California San Diego, La Jolla, California, United States of America; 3 Moores Cancer Center, University of California San Diego, La Jolla, California, United States of America; 4 Illumina Inc., San Diego, California, United States of America; The University of Hong Kong, Hong Kong

## Abstract

The ability of nicotine to enhance the malignancy of cancer cells is known; however, the possibility that nicotine could regulate a cancer stem cell phenotype remains to be well-established. In this study we sought to determine whether long-term exposure to nicotine could promote cancer stem cell-like properties in two head and neck squamous cell carcinoma cell lines, UMSCC-10B and HN-1. Nicotine treatment induced epithelial-to-mesenchymal transition (EMT) in both cell lines by repressing E-cadherin expression, and led to the induction of stem cell markers Oct-4, Nanog, CD44 and BMI-1, which was reversed upon ectopic re-expression of E-cadherin. Nicotine-treated cells formed spheres at a higher efficiency than non-treated cells, formed larger tumors when injected into mice, and formed tumors with 4-fold greater efficiency compared to control cells when injected at limiting doses. Consistent with previous literature, nicotine-treated cells demonstrated a greater capacity for survival and also a higher tendency to invade. Comparison of microRNA profiles between nicotine and control cells revealed the upregulation of miR-9, a repressor of E-cadherin, and the downregulation of miR-101, a repressor of EZH2. Taken together, these results suggest that nicotine may play a critical role in the development of tobacco-induced cancers by regulating cancer stem cell characteristics, and that these effects are likely mediated through EMT-promoting, microRNA-mediated pathways. Further characterization of such pathways remains a promising avenue for the understanding and treatment of tobacco-related cancers.

## Introduction

Current theory suggests that cancers are maintained by a subpopulation of tumor initiating cells, or cancer stem cells (CSC). Such a cell has the capacity to give rise to whole tumors due to two fundamental properties: the ability to self-renew, and the ability to differentiate into multiple cell types [1], [2]. Critical genes for self-renewal and pluripotency in human embryonic stem cells, such as Oct-4 and Nanog, are likewise elevated in cancer stem cells [3], [4]. In addition, the surface glycoprotein CD44 and oncogene BMI1 have been identified as key markers of the CSC subpopulation in breast cancer and head and neck squamous cell carcinoma (HNSCC) [5]. When compared to CD44- cells, CD44+ cells form tumors in mice much more efficiently, show increased invasion, and exhibit markedly higher resistance to chemotherapy drugs, all hallmarks of cancer stem cells [5]–[7]. In addition, cancer stem cells form spheres in non-adherent conditions in greater size and number than differentiated cancer cells [7], [8].

It is generally proposed that cancer stem cells originate either from adult stem cells that have lost control of proliferation, or progenitor cells that have acquired the ability to self-renew. However, several studies seem to support the theory that CSCs arise from differentiated tumor cells that have undergone a process of dedifferentiation to become more stem-like. Most notably, differentiated breast cancer cells that were forced to undergo epithelial-to-mesenchymal transition (EMT) were found to exhibit a more stem cell-like phenotype [9], [10]. In addition, retinal pigment epithelial cells were found to undergo a program of transdifferentiation accompanied by EMT following retinal detachment [11].

EMT is a developmental program characterized by loss of cell adhesion, increase in cell mobility, repression of E-cadherin, and upregulation of mesenchymal markers such as N-cadherin and vimentin. EMT can be induced either by the activation of the transcription factors Snail and Twist, or by repression of E-cadherin [12]. Due to its effects on cell adhesion and mobility, EMT is often activated during cancer invasion and metastasis [13], [14].

Approximately 47,000 new cases of head and neck squamous cell carcinoma (HNSCC) arise in the US each year [15]. Statistics show that tobacco use and alcohol abuse are the primary risk factors for HNSCC, associated with over 80% of documented cases [16]. Despite the well-established link between tobacco use and cancer, there is lack of understanding of the molecular mechanisms that govern tobacco-induced carcinogenesis. The majority of studies to date have focused on DNA repair or tumor suppressor pathways in response to tobacco use, while little is known about the effect of tobacco on pathways that regulate stem cell self-renewal and pluripotency.

Studies have revealed that nicotine, the major addictive component of tobacco, promotes proliferation, invasion, and angiogenesis in tumor cells while inhibiting apoptosis [17], [18]. More intriguingly, nicotine has also been shown to induce EMT in breast and lung cancer [19]. It was recently shown that tobacco smoke can promote lung tumorigenesis by triggering inflammation, which was long believed to play a critical role in cancer development [20]. Correspondingly, nicotine exposure has been shown in several cases to lead to the activation of NF-kappaB, a key regulator of inflammatory responses [21]–[23]. From these lines of evidence it is clear that nicotine can help promote cancer development; however, its role along with that of tobacco use in general remains largely uninvestigated in context of the cancer stem cell model. Previous studies have shown that nicotine upregulates FOXM1, an oncogene and stem cell gene [24] and that it is capable of increasing the proportion of ALDH+ breast cancer cells [25]. However, neither of these studies investigated the ability of nicotine to increase sphere formation in vitro or tumor-forming efficiency at limiting dilutions in vivo, arguably some of the most important metrics in determining cancer stem cell phenotype. The relationship between nicotine and classical stem cell genes such as CD44, BMI-1, Oct-4 or Nanog, is also unknown. In this study, we sought to determine whether nicotine, through activation of EMT, could alter the phenotype of cancer cells into a more stem cell-like state. We first showed that nicotine indeed promotes EMT in HNSCC cell lines. We then used a combination of gene expression and functional assays to show whether the cells developed properties of cancer stem cells after exposure to nicotine, and whether these properties were mediated via EMT. Most importantly, we investigated whether nicotine could increase the tumor-initiating capability of HNSCC cells in vitro, solidifying its role in promoting the cancer stem cell phenotype. The results of these experiments are essential to a better understanding of the development and progression of tobacco-induced cancer from a stem cell model perspective.

## Materials and Methods

### Cell Culture and Nicotine Treatment

The HNSCC cell lines used were UMSCC10B, a gift from Dr. Tom Carey, University of Michigan and HN-1, a gift from Dr. J.S. Gutkind, National Institute for Dental and Craniofacial Research. HN-1 was derived from a tongue squamous carcinoma [26]. UMSCC10B was derived from a metastatic lymph node from a different patient [27]. Cells were cultured in low glucose DMEM supplemented with 10% fetal bovine serum, 2% L-glutamine and 2% pen-strep. For nicotine treatment, an appropriate amount of nicotine hemisulfate salt solution (Sigma-Aldrich, St Louis, MO) was directly added to the culture dish. The nicotine along with the medium was replaced every three to four days, and cells were passaged at 90% confluence. Cells were not serum-starved for any experiments in this paper.

### E-cadherin Transfection

In order to test whether the expected increase in stem cells gene expression is mediated via induction of EMT, E-cadherin was transiently transfected into these cells using Lipofectamine 2000 (Invitrogen, Carlsbad, CA), following the manufacturer’s instructions. The vector used in this study was based on the pWZL-blast backbone. Cells were harvested 72 hours later and qRT-PCR was performed to verify the induction of e-cadherin as well as determine the expression of genes of interest.

### Quantitative RT-PCR

Cells were harvested two days after being passaged at about 70–80% confluence. Total cell lysate was collected and RNA was extracted using an RNeasy kit (Qiagen). cDNA was synthesized using Superscript III Reverse Transcriptase (Invitrogen, Carlsbad, CA) as per the manufacturer’s instructions. Real-time PCR reaction mixes were prepared using Power SYBR Green (Applied Biosystems, Foster City, CA), and run on the 7300 Real-time PCR System (Applied Biosystems) using the following program: 95°C for 10 min, 95°C for 30 s, and 60°C for 1 min, for 40 cycles. Results were analyzed using the ddCt method. Experiments were done in technical triplicates and were repeated at least twice independently. GAPDH gene expression was measured as endogenous control. Primers were custom ordered (Eurofins MWG Operon, Huntsville, AL) using the following sequences: Snail forward 5′- TCTGAGTGGGTCTGGAGGTG-3′, reverse 5′- CTCTAGGCCCTGGCTGCTAC-3′; Twist forward 5′- GGGCCGGAGACCTAGATGTCATTG-3′, reverse 5′- GAATGCAGAGGTGTGAGGATGGTG-3′; GAPDH forward 5′-CTTCGCTCTCTGCTCCTCC-3′, reverse 5′-CAATACGACCAAATCCGTTG -3′. N-cadherin forward 5′- AGCTTCTCACGGCATACACC-3′, reverse 5′- GTGCATGAAGGACAGCCTCT-3′. Vimentin forward 5′- GGAAATGGCTCGTCACCTTCGT-3′, reverse 5′- AGAAATCCTGCTCTCCTCGCCT-3′. Oct-3/4 forward 5′- GCAAAGCAGAAACCCTCGTGC-3′ reverse 5′- ACCACACTCGGACCACATCCT-3′. Nanog forward 5′- GATTTGTGGGCCTGAAGAAA-3′ reverse 5′- TTGGGACTGGTGGAAGAATC-3′. CD44 forward 5′- ACACCACGGGCTTTTGACCAC-3′ reverse 5′- AGGAGTTGCCTGGATTGTGCTTG-3′. BMI-1 forward 5′- TCCACAAAGCACACACATCA-3′ reverse 5′- CTTTCATTGTCTTTTCCGCC-3′.

### Matrigel Invasion Assay

Invasion of 10B and HN-1 cells was measured using a Matrigel invasion assay (Becton Dickinson, Bedford, MA). Transwell inserts of 8 µm pore size were coated with a final concentration of 1 mg/mL of Matrigel in cold serum-free DMEM. Cells were trypsinized, and 500 uL of cell suspension (1×10^5^ cells/mL) were added in triplicate wells. The lower chamber of the transwell was filled with 750 µl of culture media containing 0.5% serum as a chemoattractant, along with the treatment of nicotine and allowed to incubate at 37°C for 48 hours. Invading cells on the lower surface that passed through the filter were fixed and stained using crystal violet in gluteraldehyde and photographed. The number of the stained nuclei was counted in a predetermined and consistent section of each well.

### Cell Survival (Clonogenic) Assay

In clonal growth assays all populations were plated in triplicate at 500 cells per 60×15 mm culture plate and cultured in a Mitomycin-C treated J2-3T3 feeder layer with media supplemented with 0.5% fetal bovine serum. Colonies were fixed and stained with a crystal violet/formalin solution and counted after 7–10 days using AlphaImager 2200 and 1220 software (Alpha Innotech Co., San Leandro, CA) such that colonies containing at least 50 cells will be counted as positive.

### Western Blotting

Nicotine-treated 10B and HN-1 were plated in complete medium and grown as normal until they reached 80% confluence. Cells were lysed following 2 days after the last nicotine treatment. Cell lysates were separated on a 4–12% SDS-polyacrylamide protein gel (Invitrogen, Carlsbad, CA, USA) and transferred onto a nitrocellulose membrane. The membrane was blocked overnight at 4°C with 5% BSA in TBS-T (TBS with 0.1% Tween-20) and incubated with primary antibodies at a 1∶1,000 dilution. After a 3×10 minute wash with TBS-T, the membrane was incubated with the appropriate secondary antibody at 1∶10,000 dilution at room temperature. After three additional washes, the protein-antibody complexes were detected using enhanced chemiluminescence (Pierce, Rockford, IL).

### Immunofluorescence

10B and HN1 cells were cultured on cover slips under the nicotine and non-nicotine conditions previously stated. The cells were fixed with 4% paraformaldehyde and blocked in goat serum in Dulbecco’s phosphate buffered saline at room temperature prior to incubation with mouse monoclonal to anti-human vimentin (Sigma Aldrich, St. Louis, MO). Cells were then incubated with a goat anti-mouse FITC conjugated secondary antibody (Chemicon, Temecula, CA) and counterstained with DAPI. Finally, SlowFade Gold antifade reagent (Invitrogen, Carlsbad, CA) was used to mount the cover slips onto slides. Fluorescent images were obtained at 40X using Leica DMIRE2 inverted fluorescence microscope and computer program Simple PCI was used for image capture.

### Sphere Formation Assay

Cells were seeded in a 24-well low adhesion plate (Corning Inc, Lowell, MA) at a density of 1500 cells/well and an initial volume of 500 ul. Spheres were grown in DMEM F12 medium (Gibco) supplemented with Vitamin B27, 20 ng/ml EGF, 20 ng/ml FGF, and 4 ug/ml of heparin. EGF and FGF were replenished every three days and 500 ul of fresh media with or without nicotine was added every four days. Aside from the addition of heparin, cells were agitated daily to minimize clumping. After two weeks, cells were counted and photographed.

### Tumorigenicity Assay in SCID Mice

Control and nicotine-treated HN-1 cells were trypsinized, pelleted and resuspended at the appropriate concentration in a 1∶10 solution of matrigel (Sigma-Aldrich, St. Louis, MO). 200 ul of cells (5000 or 200,000 cells) were then injected subcutaneously into SCID mice. After 12 weeks, the mice were sacrificed and the tumors were compared between control and nicotine-treated samples. Mice were not administered nicotine throughout the experiment. All animal work was approved and conducted in accordance with UCSD IRB policy and procedures (protocol #S07410).

### MicroRNA profiling

MicroRNA was isolated from treated and non-treated cells using the mirVana miRNA isolation kit (Ambion, Austin, TX), following the manufacturer’s instructions. Samples were run on the Illumina MicroRNA Array Profiling platform [28]. Analyses were performed using BRB-ArrayTools developed by Dr. Richard Simon and BRB-ArrayTools development team. Clustering algorithms were performed by Cluster 3.0 and visualized with TreeView (Eisen Lab, Stanford University).

## Results

### Nicotine Treatment Induces Epithelial-to-mesenchymal Transition in HNSCC Cell Lines

**Figure 1:**
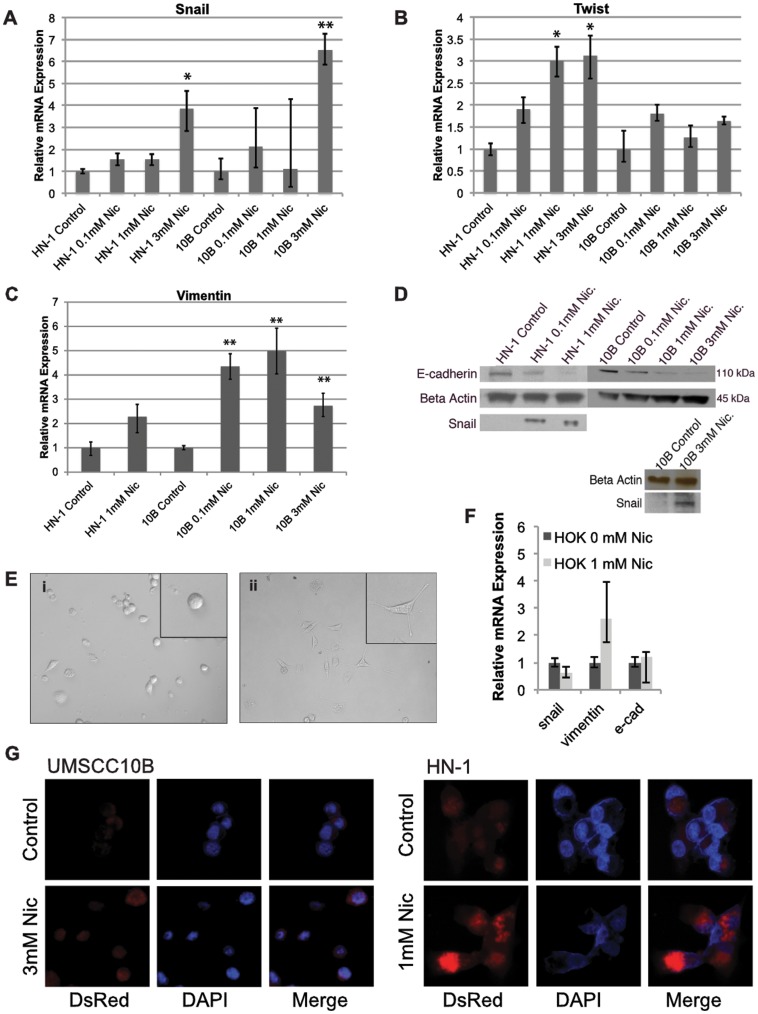
Long term treatment of nicotine alters mRNA levels of epithelial to mesenchymal transition expression in head and neck squamous cell carcinoma cell lines UMSCC10B and HN-1. A) Snail, B) Twist, C) Vimentin were all induced corresponding to an increased EMT phenotype. D) Western blot showing increased EMT expression in Snail and decreased E-cadherin in UMSCC10B and HN1 following long-term exposure to nicotine. E) Nicotine-treated HN-1 cells (ii) had a more mesenchymal morphology compared to non-treated cells (i). F) Effect of nicotine on expression of EMT markers in normal oral keratinocytes. G) EMT is confirmed by immunofluorescence, with nicotine treated cells displaying higher expression of the mesenchymal marker vimentin. All images were photographed under 40X. *p<0.05, **p<0.01.

In the process of determining whether nicotine can promote a more stem cell-like phenotype, we first assessed its ability to induce epithelial-to-mesenchymal transition in the HNSCC cell lines UMSCC10B and HN-1. 10B and HN-1 cells were cultured in the presence of 3 mM and 1 mM nicotine, respectively, for 6 weeks. Such dosages are within physiological limits of nicotine concentrations found in the saliva of tobacco users [18]. In addition, long-term treatment was intended to mimic the conditions of carcinogenesis in habitual tobacco users. At 6 weeks, expression of the EMT markers Snail, Twist, and Vimentin were assayed by qRT-PCR in both cell lines (Fig. 1A, 1B, 1C). At a sufficient dose of nicotine (which is cell-line dependent), nicotine-treated cells expressed significantly higher levels of mesenchymal markers and lower levels of epithelial markers, suggesting that long-term nicotine treatment is capable of inducing EMT in these two HNSCC cell lines. To further confirm the acquisition of an EMT phenotype, western blots were performed to determine the protein levels of Snail and E-cadherin (Fig. 1D). Nicotine-treated HN-1 cells displayed a fibroblast-like morphology, indicating mesenchymal phenotype (Fig. 1E) which was further confirmed by an immunofluorescence experiment showing increased expression of vimentin (Fig. 1G). Together, these results show that nicotine induces EMT under our experimental conditions, and are consistent with previous studies performed on breast and lung cancer. As a control, we also performed an experiment to test the effect of nicotine on EMT markers in normal oral keratinocytes (Fig. 1F).

### Nicotine-treated Cells Exhibit Higher Levels of Stem Cell Markers

**Figure 2:**
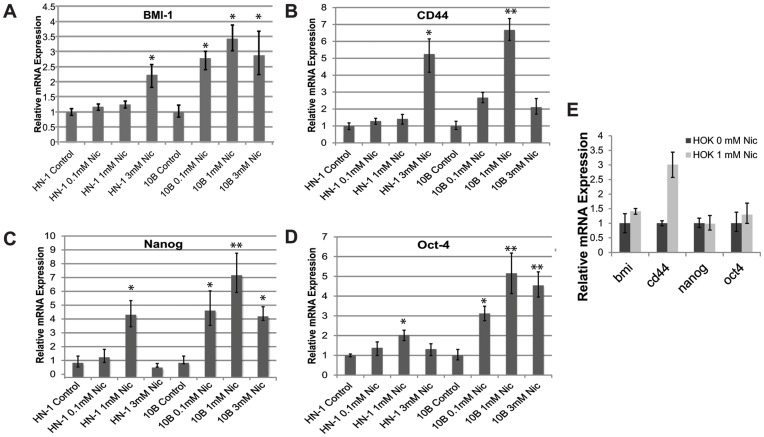
Long term treatment of nicotine increases mRNA expression of stem cell genes in head and neck squamous cell carcinoma cell lines UMSCC10B and HN-1. A) CD44, B) Bmi1, C) Oct4, D) Nanog. E) Effect of nicotine on expression of stem cell markers in normal oral keratinocytes. *p<0.05, **p<0.01.

CD44 and BMI-1 are critical markers of self-renewal in HNSCC, and their expression has been implicated in tumorigenic potential as well as metastatic ability. Oct-4 and Nanog play essential roles in pluripotency and self-renewal in human embryonic stem cells. We therefore reasoned that the increased expression of these markers is indicative of a more stem cell-like state. The expression of these markers was compared between control and nicotine-treated cells using qRT-PCR (Fig. 2A–D). The expression of stem cell markers was upregulated in nicotine-treated cells, indicating possible regulation of CSCs by nicotine. As a control, we also performed an experiment to see whether nicotine could induce similar changes in normal human keratinocytes (Fig. 2E).

### Nicotine Exposure Increases Survival, Sphere Formation, Tumor Size, and Multipotency

**Figure 3:**
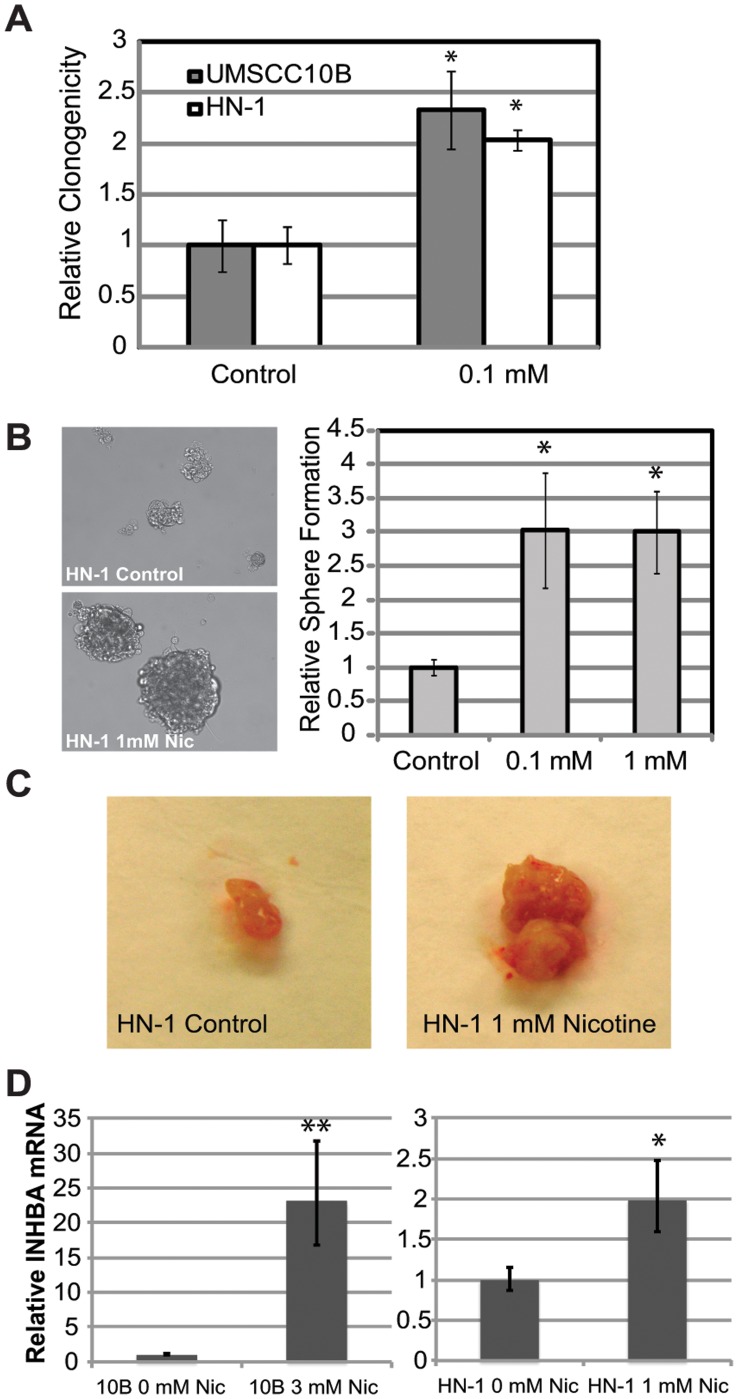
Increased cell survival, sphere formation and tumor size after exposure to nicotine. A) Nicotine enhanced the ability of 10B and HN-1 cells to form colonies under low serum conditoins, suggesting increased survival capacity. B) Photos on left are representative images of spheres formed by nicotine-treated or control HN-1 cells. Graph on right indicates the significant increase in number of spheres formed by nicotine-treated HN-1 cells compared to untreated cells. C) Representative photos comparing tumor sizes between 1 mM-nicotine-treated HN-1 cells and untreated HN-1 cells. D) Relative expression of the multipotency regulator INHBA between nicotine-treated cells and control cells. *p<0.05, **p<0.01.

Nicotine treated cells exhibited a greater capacity to survive, as determined by a clonal growth assay (Fig. 3A). Both CSCs and normal stem cells have been demonstrated previously to form spheres at much higher efficiency compared to differentiated cells [29]. We sought to determine the effect of long term nicotine exposure on sphere formation, as it could be an indicator of a CSC-like phenotype. Nicotine-treated HN-1 cells were seeded in a 24-well low adhesion plate format (Corning Inc, Lowell, MA) at a density of 1500 cells per well. After two weeks, spheres were counted. We found that long term nicotine treated cells formed a greater number of spheres compared to control (Figure 3B right). In addition, nicotine exposed cells formed spheres larger in diameter compared to control cells, which seemed to form aggregates rather than true spheres (Fig. 3B left). This shows that nicotine-exposed cells have a higher capacity to self-renew and proliferate in suspension, which is a critical property of both normal and cancer stem cells [29]. To perform a preliminary survey on the effect of nicotine on tumor formation, we injected both control and 1 mM nicotine-treated HN-1 cells (500,000 cells) into SCID mice and observed that nicotine-treated cells formed significantly larger tumors compared to control cells (Figure 3C). To evaluate multipotency, we looked at the effect of nicotine treatment on the expression of INHBA, a regulator of multipotency in mesenchymal stem cells (Figure 3). This experiment revealed that nicotine upregulates INHBA, indicating increased multipotency. Together these results suggest that long-term exposure to nicotine could promote the acquisition of some functional properties of cancer stem cells.

### Nicotine-treated Cells are More Invasive Compared to Control Cells

**Figure 4:**
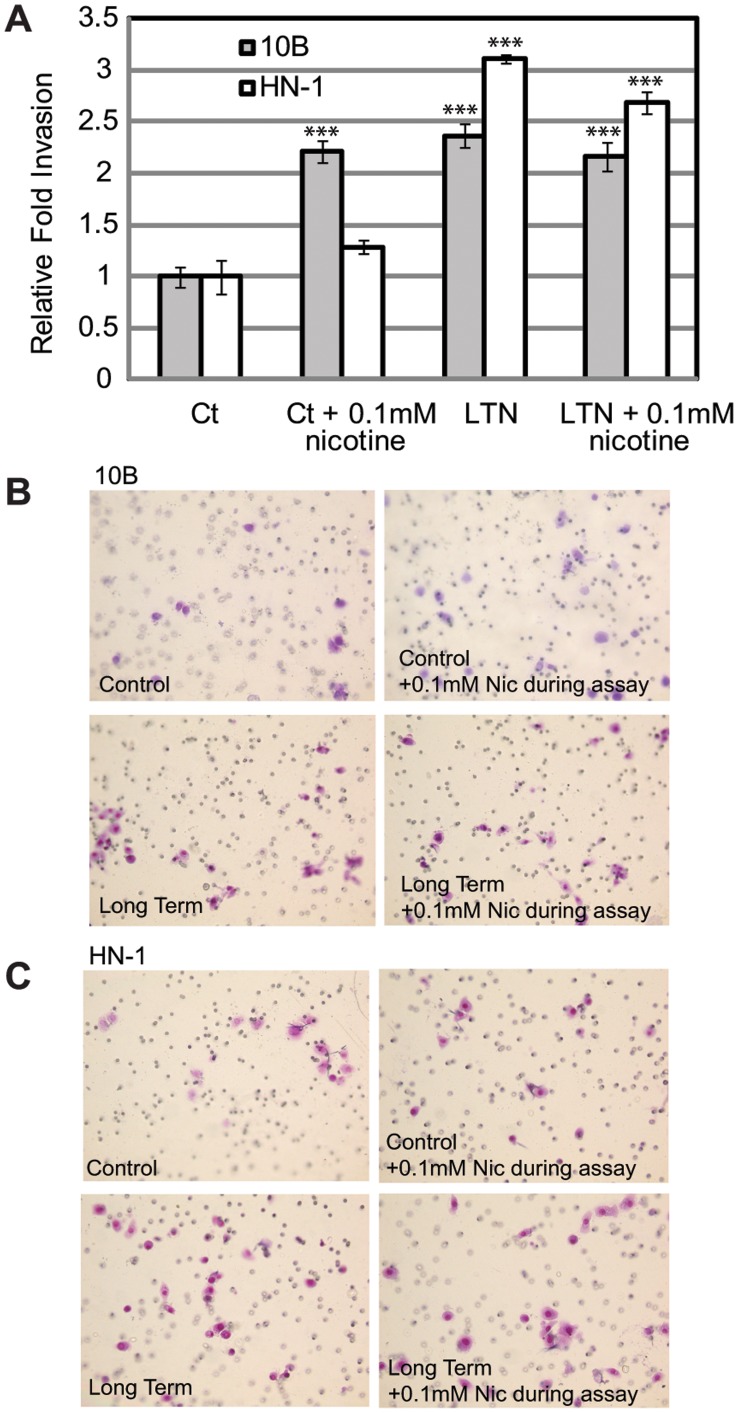
Long-term nicotine exposure increases invasiveness of 10B and HN-1. A) Bar graph indicating the results of a matrigel invasion assay. B) Representative wells of invaded 10B cells. Permutations include control, control with addition of 0.1 mM nicotine during the invasion assay, cells treated long term in 0.1 mM nicotine without addition of nicotine during assay, cells treated long term in 0.1 mM nicotine with addition of 0.1 mM nicotine during assay. C) Representative wells of invaded HN-1 cells, with same permutations as above. *p<0.05, **p<0.01, ***p<0.001.

CSCs are characteristically more invasive compared to normal tumor cells. To determine whether long term nicotine treatment results in a higher level of cellular invasion, we performed a matrigel invasion assay comparing treated 10B and HN-1 to control 10B and HN-1 cells. Our results showed 2–3 fold higher invasion in nicotine treated cells, which is possibly a consequence of the EMT induced by nicotine (Figure 4).

### Nicotine Enhances In Vivo Tumor Growth and Tumorigenicity

**Figure 5:**
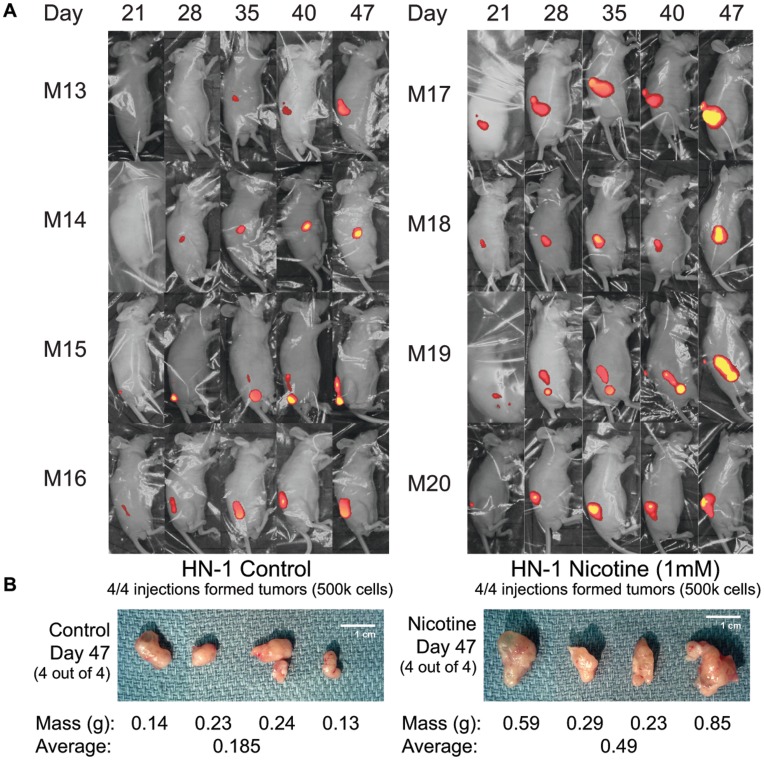
Long-term nicotine exposure increases size of tumors formed in nude mice. A) Fluorescent whole-body imaging of nude mice each injected with 500 k of either control or 1 mM-nicotine-treated HN-1 cells overexpressing RFP. B) Photographs and weights of tumors dissected from mice 47 days post-injection.

**Figure 6:**
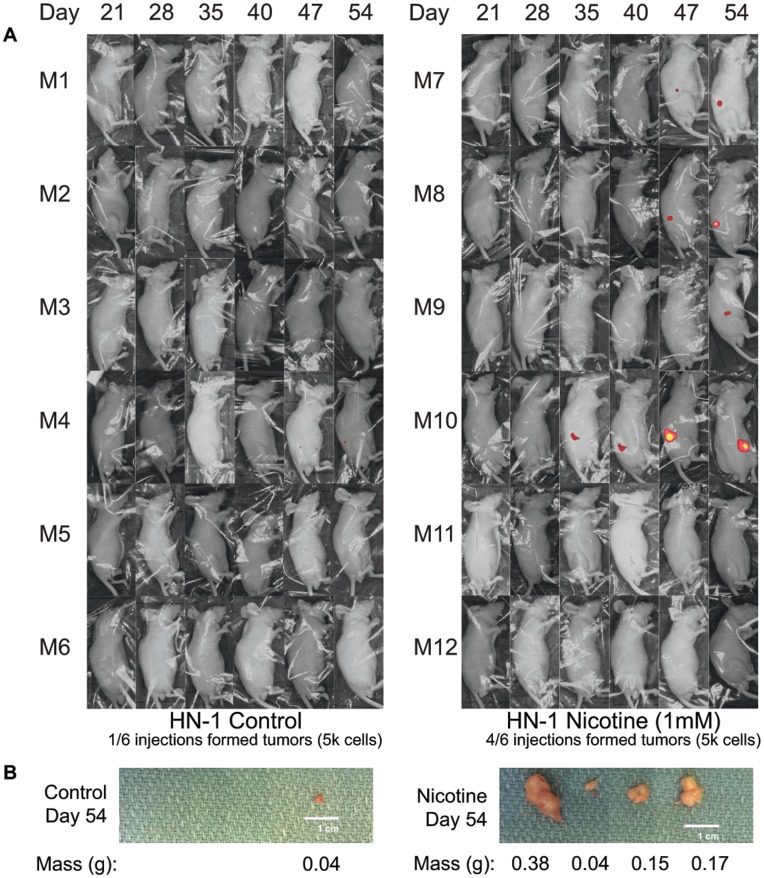
Long-term nicotine exposure increases tumorigenicity. A) Fluorescent whole-body imaging of nude mice each injected with 5 k of either control or 1 mM-nicotine-treated HN-1 cells overexpressing RFP. B) Photographs and weights of tumors dissected from mice 54 days post-injection.

To confirm and strengthen our preliminary findings in mice, we performed two sets of mouse experiments, one demonstrating that nicotine-treated cells form bigger tumors in nude mice compared to control cells, and that nicotine-treated cells also form tumors more efficiently at limiting dilutions. HN-1 control and HN-1 1 mM long term nicotine cells were first made to overexpress red fluorescent protein (RFP) using a viral-based vector. After an RFP-expressing population was confirmed by fluorescent microscopy, these cells were injected subcutanouesly at two different doses (5 k and 500 k) into the flanks of nude mice. Mice were not administered nicotine throughout this experiment. Mice that were injected with 500 k cells were sacrificed on the 47^th^ day, while those injected with 5 k cells were sacrificed a week later. Results indicated that at a dose of 500 k cells, nicotine-treated HN-1 cells formed significantly larger tumors, with an average weight of 0.49 g compared to 0.185 g for control cells (Figure 5). At a dose of 5 k cells, nicotine-treated HN-1 cells were 4 times more tumorigenic compared to control cells, forming tumors at 4 out of 6 of the injection sites versus 1 out of 6 for control (Figure 6).

### Long-term Nicotine Exposure Results in Differential Expression of Critical miRNAs

**Figure 7:**
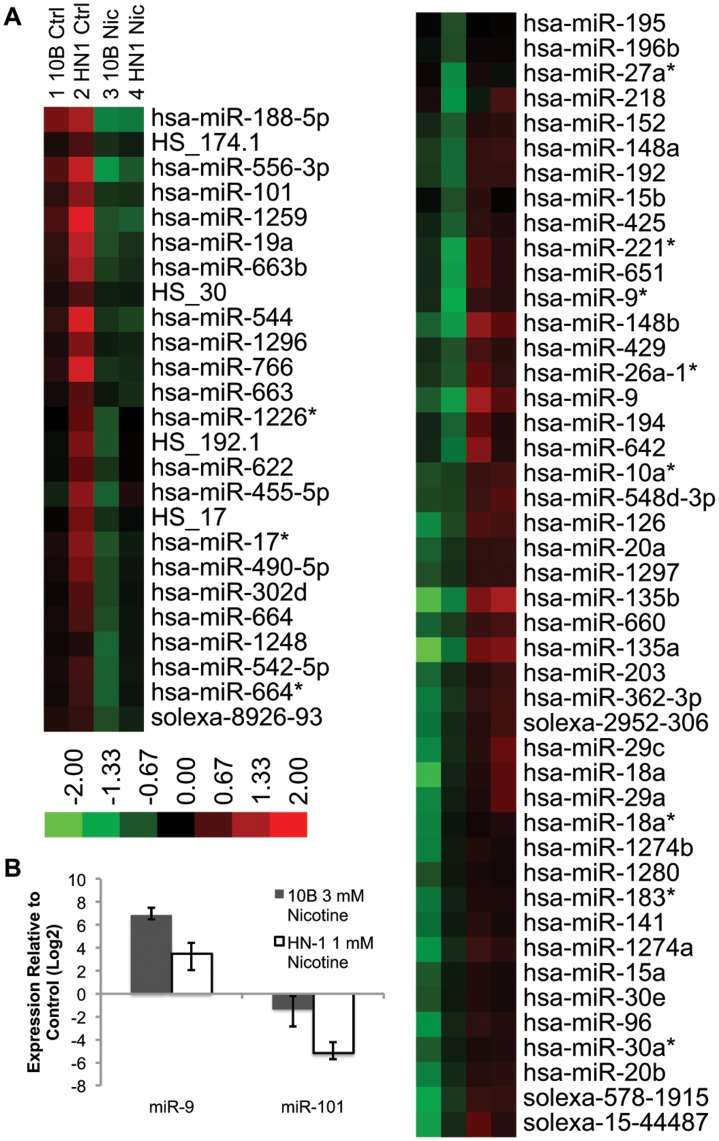
MicroRNA profile of nicotine-treated vs non-treated cells. A) Log expression values are computed based on fold expression from the median value within each row. Genes were excluded based on the criteria of having at least 20% of expression values showing a 1.5 fold increase or decrease relative to the median. Downregulated genes that clustered with miR-101 and upregulated genes that clustered with miR-9 were magnified within the figure. B) qPCR validation of miR-9 and miR-101 expression.

To address the possibility that the effects of nicotine treatment are miRNA-mediated, we performed an array analysis of global miRNA expression using Illumina’s MicroRNA Profiling assay. A handful of miRNAs were shown to be significantly up- or down-regulated in both cell lines after long-term exposure to nicotine (Figure 7A). These included several genes which have been shown previously to play roles in EMT and other oncogenic properties. Upregulated genes included miR-9, which was previously shown to inhibit cancer metastasis by suppressing e-cadherin [30], and miR-221 [31], which contributes to liver tumorigenesis. miR-135a/b was also highly upregulated and is known to be a potent inducer of Wnt pathway activity [32]. Downregulated miRNAs included miR-101, which is a tumor suppressor and inhibitor of EZH2 [33]. The expression of these two microRNAs were confirmed and validated by qPCR (Figure 7B).

### Transfecting e-cadherin in Nicotine-treated Cells Partially Reverts the Previously Observed Induction of Stem Cell and EMT Markers

**Figure 8:**
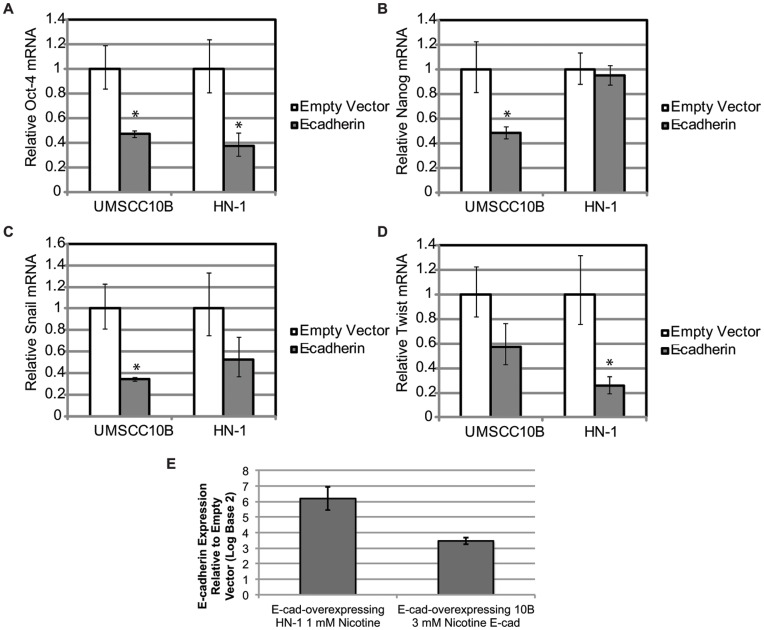
Overexpression of E-cadherin decreases expression of (A) Oct-4, (B) Nanog, (C) Snail, and (D) Twist, indicating a reversal in phenotype induced by long term nicotine treatment. Expression values are compared against an empty vector control. E) indicates the overexpression level of e-cadherin.

To determine whether the induction of stem cell markers were conferred via EMT signaling, we transfected mouse e-cadherin into long term nicotine treated 10B and HN-1. Cells transfected with e-cadherin showed reduction of these stem cell markers (Figure 8). These results suggest that nicotine-induced expression of pluripotency markers Oct-4 and Nanog are at least partially mediated through EMT. It remains to be clarified whether there exists a pathway aside from EMT or a more direct manner in which nicotine may modulate the expression of Oct-4 and Nanog. The overexpression of e-cadherin was greater than ten-fold in all of these experiments.

### Reversal of Nicotine-induced Gene Expression Occurs Following Withdrawal of Nicotine

**Figure 9:**
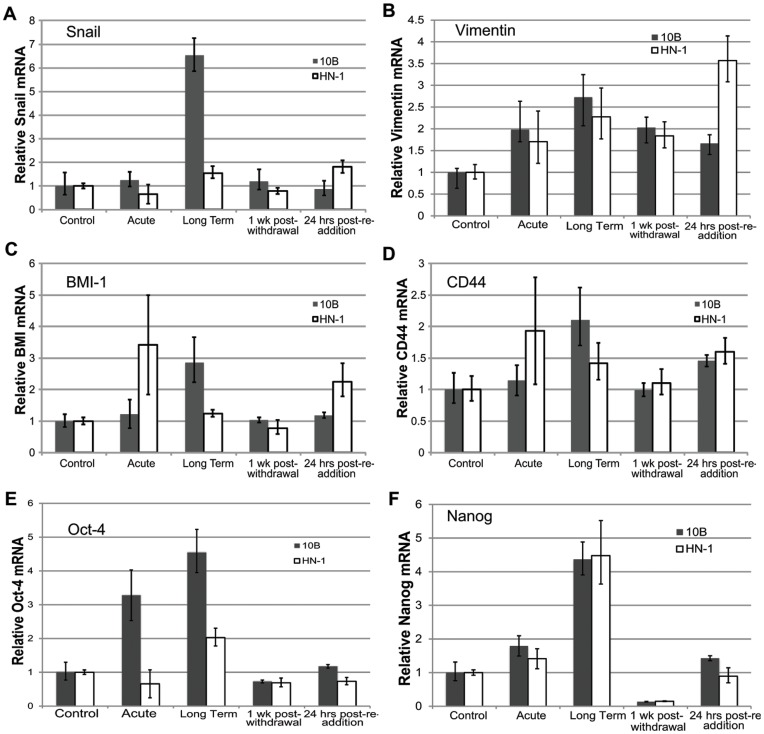
Effect of nicotine withdrawal and subsequent reintroduction on long term nicotine-treated cells. Bar graphs showing expression of (A) Snail, (B) Vimentin, (C) BMI1, (D) CD44, (E) Oct-4, (F), Nanog under various conditions.

To determine whether the effects of nicotine could be reversed by withdrawal of nicotine, we looked at expression of stem cell and EMT markers in nicotine-treated cells after a week-long withdrawal from nicotine. Our results revealed that the expression levels of these markers reverted to basal levels within one week of nicotine withdrawal (Figure 9). Re-addition of nicotine for 24 hours slightly induced these markers above basal levels in some cases, but for the most part did not elicit a significant response, suggesting that long-term nicotine treatment does not confer a “memory” effect whereby cells become more sensitized to future nicotine exposure.

## Discussion

Here we have shown that long-term nicotine exposure generates cells with properties that are reminiscent of cancer stem cells. Our findings may be important for understanding tobacco-induced carcinogenesis in the context of the cancer stem cell paradigm. Knowing that nicotine or tobacco enables the acquisition of CSC-like properties, one can speculate on the implications of this finding in regard to tobacco-induced carcinogenesis and appropriate treatment modalities. First, most smoking-cessation programs currently rely on the chronic administration of nicotine. Our findings suggest that such treatments can potentially increase the risk for cancer by promoting self-renewal and EMT in premalignant cells even after tobacco use has stopped. Second, the potential ability of nicotine to bring about a malignant stem cell state implies that the CSC model is relevant in tobacco-induced carcinomas. Because nicotine induces a subpopulation of CSCs, treatments specifically aimed at targeting CSCs could be both justified and necessary in the treatment of tobacco-induced cancers. While nicotine typically inhibits apoptosis, it would be interesting to determine whether long-term nicotine exposure can increase cell sensitivity towards CSC-specific inhibitors, such as salinomycin or metformin [34], [35]. Third, the way in which nicotine contributes to the development of cancer can potentially be described by a model in which vital tumor suppressors are first rendered inoperative by various carcinogens found in tobacco. In some fashion, this event would greatly potentiate the ability of nicotine to activate EMT or other dedifferentiation pathways and drive the development of the cancer into an aggressive and metastatic phenotype. Another possibility is that if given enough time, nicotine can act upon somatic stem cells or progenitor cells to induce a cancer stem cell phenotype perhaps through the persistent activation of inflammatory cascades and through sustained oxidative stress.

We have shown that overexpressing E-cadherin in nicotine-treated cells partially reverses the induction of the stem cell genes Oct-4 and Nanog, suggesting that the stem cell properties conferred by nicotine are mediated at least in part by EMT. Our findings seem to be consistent with the observations of Dasgupta et al, who suggested that nicotine-induced EMT is primarily mediated through the repression of ECM proteins such as E-cadherin. Future experiments must characterize the mechanisms by which nicotine represses E-cadherin and induces EMT, as well as other targets of nicotine which may contribute to the acquisition of a CSC-like state. Our profiling experiments revealed a set of differentially expressed microRNAs between treated and non-treated cells, suggesting a role of miRNAs in mediating the effects of nicotine. Most notably, miR-9, which was upregulated in nicotine-treated cells, has been shown to promote metastasis in breast cancer by repressing E-cadherin [30]. miR-9 has also been shown to control the migration and proliferation of progenitor cells derived from hESCs [36]. The roles of miR-9 and other differentially expressed miRNAs remain to be fully established in the context of nicotine-induced EMT, and should be addressed by future experiments.

Studies indicate that epigenetic mechanisms may play an essential role in regulating EMT [37]–[39]. However, little is currently known about the effect of nicotine on expression and activity of DNA methyltransferases and HDACs, although this information may be critical in describing the mechanisms of our findings. Possible targets include DNMT1, whose expression has been linked to the repression of E-cadherin [40], [41]. In addition, E-cadherin has been shown to be downregulated by a transcriptional repressor complex consisting of Snail, HDAC1 and HDAC2 [42].

Aside from EMT, pathways that are induced by nicotine and may also contribute to the acquisition of stem cell characteristics include the well-studied PI3K/Akt and MAPK signaling pathways. Nicotine was found to reduce the senescence of endothelial progenitor cells by increasing telomerase activity via Akt signaling [43]. In addition, the anti-apoptotic effects of nicotine have been shown to be partly mediated by PI3K/Akt and MAPK in nasal epithelial cancer [44]. It is possible that nicotine, which has been shown to target NF-kappaB [21], may also work through pro-inflammatory pathways to promote stem cell properties. Takahashi et al recently described a model of lung tumorigenesis in which tobacco smoke acts as a tumor promoter, causing increased proliferation of chemically (NNK) and genetically (K-Ras activation) induced lung cancer cells *in* *vivo* through IKKβ- and JNK1-mediated inflammatory signaling. As tobacco smoke comprises a myriad of agents, it would be useful from a molecular perspective to examine the isolated role of nicotine in that context.

Removal of nicotine seems to allow the expression of stem cell and EMT markers to revert to their original levels within a week, suggesting that maintenance of EMT and stem cell characteristics may require sustained exposure to nicotine. There is also little evidence of sensitization towards nicotine that is brought on by long-term nicotine treatment, as acute reintroduction of nicotine to cells that have undergone withdrawal did not seem to elicit a response that was significantly different from control cells. Even though the effect of nicotine on stem cell and EMT gene expression seems to wear off easily, nicotine-treated cells were still more tumorigenic in mice that did not receive nicotine throughout the course of the experiment. Thus, even the brief timeframe in which nicotine enhances the stem cell phenotype of the cancer cell may make the difference in terms of whether it is able to form a tumor.

The exact conditions required for nicotine-induced stemness have yet to be identified and future studies must determine which genetic mutations, if any, are prerequisites for nicotine to induce EMT and dedifferentiation in tumor cells. It must also be examined whether nicotine or tobacco can activate similar pathways of EMT and dedifferentiation in normal adult progenitor cells, as this would have enormous implications in cancer stem cell biology, specifically in helping to understand the origin of cancer stem cells. As of now, our findings imply that nicotine, due to its ability to regulate EMT and stem cell properties, could hold a much more essential role in cancer development than previously thought. Preliminary studies in the cancer stem cell field have shown that selective targeting of CSCs can significantly reduce the rate of tumor formation as well as metastasis in mice [35]. Further studies to characterize the pathways through which nicotine acts to promote stem cell properties could therefore greatly contribute to our understanding of the initiation and progression of tobacco-induced cancers, and may lead the way into more novel and effective treatment modalities for these diseases.
